# Primary Bone Lymphoma: A Rare Cause of Chronic Back Pain

**DOI:** 10.7759/cureus.21147

**Published:** 2022-01-12

**Authors:** Guilherme Cunha, Martim Alçada, Ana Mestre, Marta B Duarte, Filomena Roque

**Affiliations:** 1 Internal Medicine, Hospital Distrital de Santarém, Santarém, PRT; 2 Hematology, Hospital Distrital de Santarém, Santarém, PRT

**Keywords:** multifocal bone lymphoma, polyostotic lymphoma, extranodal diffuse large b-cell lymphoma, lymphoma of the bone, primary bone lymphoma

## Abstract

Primary bone lymphoma is a very uncommon malignancy, which is responsible for 3% of all bone tumors. We report a case of an 80-year-old patient with chronic back pain associated with a pathological T9 fracture. During admission, spinal cord compression with paraparesis was detected and managed with radiotherapy. After investigation, it was discovered to be caused by a primary bone lymphoma. Staging showed multiple bone lesions compatible with polyostotic lymphoma. Histopathology revealed a diffuse large B-cell lymphoma, which was treated with chemotherapy (age-adjusted R-CHOP [rituximab, cyclophosphamide, doxorubicin, vincristine, and prednisone] regimen). In this case report, imaging modalities used to diagnose and stage the disease are discussed. Traditional and new prognostic tools and treatment are also reviewed.

## Introduction

Lymphomas are cancers of B, T, and natural killer cells that originate in lymphoid tissue (nodal disease) or in tissues outside the lymph nodes (extranodal disease). The presence of Reed-Sternberg cells is one of many characteristics of Hodgkin lymphoma. All other lymphomas are classified generally as non-Hodgkin lymphomas. Extranodal disease has been described in many locations, namely craniofacial, thyroid, breast, and bone [1].

Lymphomas can affect the skeleton either as a solitary lesion or as part of disseminated disease. The definition of primary bone lymphoma (PBL) has evolved during the last few decades. The current WHO classification of Soft Tissue and Bone Tumors describes PBL as a neoplasm composed of malignant lymphoid cells, producing one or more lesions within the bone, with no lymph node involvement or other extranodal lesions [2]. While the classification of a unique bone lesion is relatively straightforward, the allocation of patients with multifocal osseous lesions (10-40% of cases) but with no lymph node or visceral involvement is not consensual. This latter group is named “multifocal osseous lymphoma” or “polyostotic lymphoma,” as suggested by the International Extranodal Lymphoma Study group in the IELSG 14 study [3].

PBL is a rare disease encompassing only 3% of all malignant bone tumors and less than 5% of all extranodal non-Hodgkin lymphomas [4].

## Case presentation

An 80-year-old active man (with a Karnofsky Performance Status scale of 100 points) presented with midline, lower back pain. His past medical history included arterial hypertension, type 2 diabetes, and osteoarthritis. His usual medication was perindopril and indapamide, sitagliptin, and glimepiride. The back pain begun insidiously six months prior, with progressive increasing intensity. The pain was dull and worse with active movement. Symptomatic medication with paracetamol and nonsteroidal anti-inflammatory drugs, previously prescribed, was insufficient for pain control. Upon questioning, he mentioned involuntary weight loss of 8 kg (10.6% of total body weight). There were no complaints of muscular weakness, paresthesia, or previous episodes of trauma (including minor events). Absence of fever, nights sweats, constipation, or urinary incontinence was noted.

On examination, the patient was alert and cooperative. An assessment of the respiratory and cardiovascular system did not reveal any abnormalities. There was no palpable lymphadenopathy hepatomegaly or splenomegaly. Visual inspection of the thoracic and lumbar spine revealed no masses or swelling.

**Figure 1:**
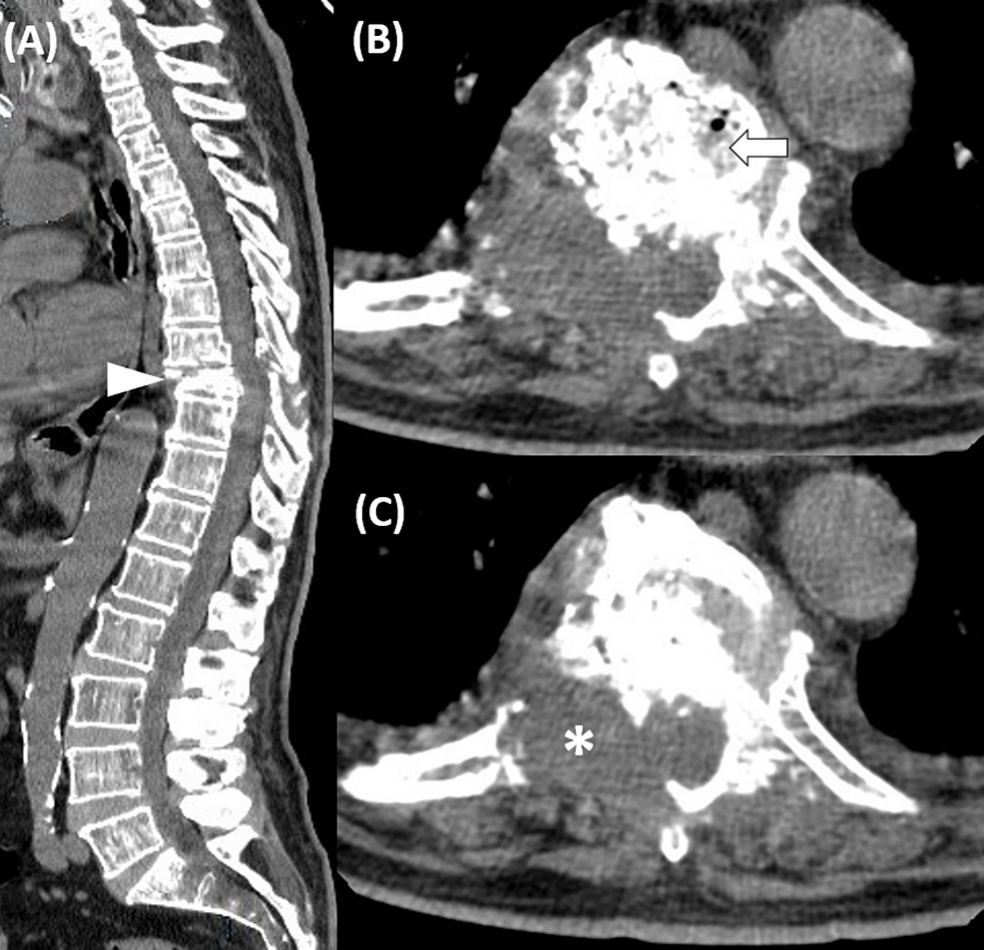
Contrast-enhanced computed tomography (A) Sagittal plane: fracture and collapse of the ninth dorsal vertebra body (arrowhead). (B) Horizontal plane: lytic lesion of the vertebral body (arrow). (C) Horizontal plane: soft tissue formation extended to the right paravertebral space as well as to the anterior epidural space (asterisk).

Blood investigations showed a hemoglobin value of 12.2 g/dL, peripheral white cell blood count of 9.3 x 10^9^/L, and platelet count of 276 x 10^9^/L. Further investigations yielded an erythrocyte sedimentation rate of 87 mm/hour (reference range 0-22 mm/hour), lactic acid dehydrogenase (LDH) of 187 U/L (reference range 100-190 U/L), alkaline phosphatase of 123 U/L (reference range 44-147 U/L), normal albumin levels, and protein electrophoresis, as well as a prostate-specific antigen value of 2.33 ng/mL (normal value: below 4.5 ng/mL). Contrast-enhanced computed tomography (CT) scan revealed a soft tissue mass in the ninth thoracic vertebrae, with lytic destruction and collapse of the vertebral body (Figure 1).

**Figure 2:**
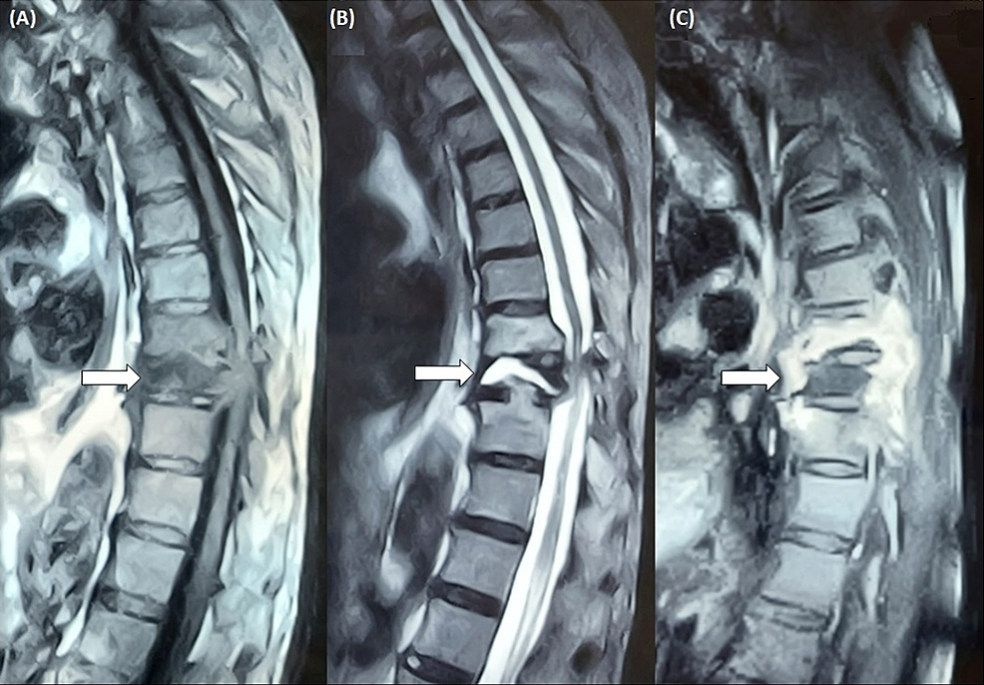
Magnetic resonance imaging of the thoracic spine (sagittal plane) (A) T1-weighted image shows a hypointensive lesion at the level of the ninth thoracic vertebra with protrusion to the spinal cavity and prevertebral space (arrow). There is collapse and sclerosis of the T9 vertebral body. (B) T2-weighted image shows hyperintensity of the same dorsal vertebra (arrow) and spinal compression. (C) T1-weighted image post-gadolinium highlights the extension of the lesion to the prevertebral planes, as well as T8 and T10 (arrow).

The patient was admitted with the diagnosis of a pathological vertebral fracture, concerning for metastatic disease. Staging CT showed no other abnormalities. On the fifth day of admission, the patient complained of bilateral lower limb weakness and hypoesthesia. Further urgent assessment using magnetic resonance imaging (MRI) revealed edema and compression of the spinal cord, in addition to extensive infiltration to the posterior arch of T8, T9, and T10 (Figure 2).

The patient underwent five sessions of decompressive radiotherapy to reduce the spinal cord pressure. However, he only recovered partially and became dependent of some assistance (Karnofsky Performance Status scale of 60 points). A CT-guided biopsy was performed. Histopathological examination revealed intense diffuse infiltration by lymphoid cells of medium to large size, with irregular nucleus and multiple nucleoli. There were numerous images of apoptosis and mitosis. In the immunohistochemical study, cells were immunoreactive for CD20, CD10, BCL-2, BCL-6, MUM1, and vimentin. Also, the proliferative index was high (approximately 90%) and compatible with diffuse large B-cell lymphoma (DLBCL) of non-germinal center B type.

**Figure 3:**
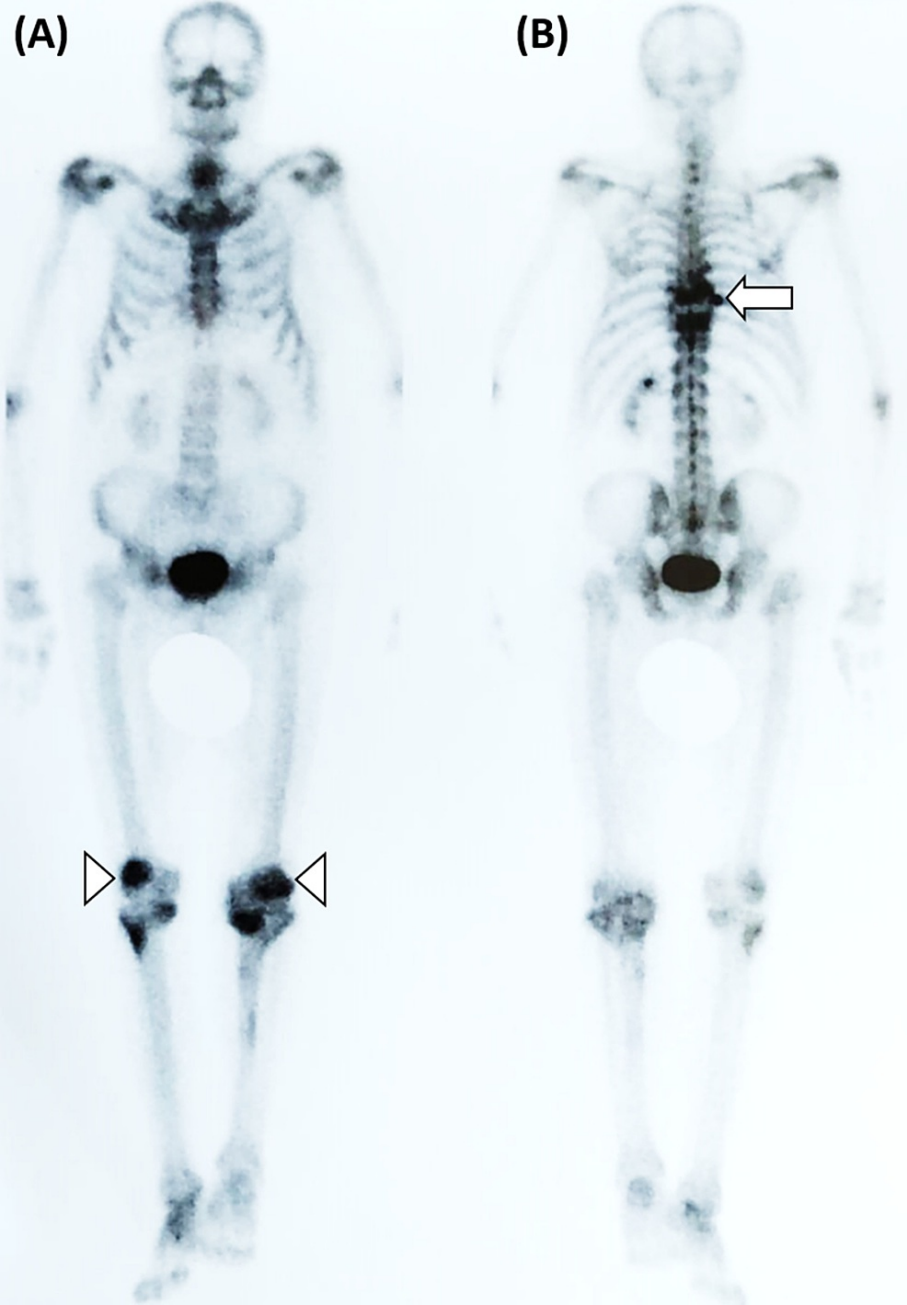
Whole-body PET scan (A) Anterior view: moderate and heterogeneous uptake in both knees of degenerative etiology (arrowheads). (B) Posterior view: high uptake areas at the level of T9 and T10 vertebras (arrow). PET, positron emission tomography

The whole-body positron emission tomography (PET) scan showed high radiopharmaceutical uptake at the level of T9 and T10 vertebra (Figure 3).

Chemotherapy with an age-adjusted R-CHOP (rituximab, cyclophosphamide, doxorubicin, vincristine, and prednisone) regimen was initiated. The patient was discharged to the outpatient hematology clinic and continued chemotherapy. The patient passed away several days later from an acute myocardial infarction.

## Discussion

Current knowledge regarding clinical characteristics, treatment, and prognostic factors of PBL is limited due to its low incidence. Available data are derived solely from case series and retrospective studies. A slight male predominance (male/female ratio of 1.5/1) has been consistently present, with a median age at diagnosis ranging from 45 to 60 years [5].

Pain is the most prevalent symptom followed by the presence of a tumor mass and a pathologic fracture [6]. The average time elapsed between the beginning of symptoms and diagnosis is eight months. Lymphomas can develop in any bone; historically, the femur was the most common location mainly in the diaphysis [7]. However, the spine and pelvis are becoming more frequent sites of PBL presentation, especially in association with pathological fractures [8]. Given our patient's increased age, the assumption of attributing pain to osteoarthritis may have led to a delay of diagnosis. Notwithstanding, the patient reported involuntary weight loss, which is a warning sign when associated with chronic back pain. Fever, night sweats, and weight loss are less frequent in PBL when compared with systemic lymphomas, and their presence may herald advanced disease.

Symptoms of spinal cord compression are the presenting features of 16% of cases [9]. In our patient, symptoms developed suddenly during admission. However, in the literature, more insidious complaints have been reported [10]. Symptoms attributed to hypercalcemia (constipation, lethargy, and somnolence) are rare.

PBL is typically associated with non-specific radiographic findings. On plain X-rays, lesions are more often lytic, but osteoblastic lesions and mixed patterns can be present. Contrast-enhanced CT is used to better define the extraosseous extension of lesions as well as cortical tumor breakthrough. MRI provides further detail on disease extension and tumor invasion, especially when involving the central nervous system. Usually, abnormal intensity areas are visible. They are hypointense on T1-weighted and hyperintense in T2-weighted imaging [11].

Functional imaging assessment with 18FDG-PET scan is used to identify high uptake lesions. The addition of 18FDG-PET to CT scan enabled the upstaging of 42% of lymphoma patients in one study [12]. The combined use of CT scan and PET scan, named 18FDG-PET-CT scan, provides concurrent functional and anatomic information. It is the image modality recommended by the Lugano classification for staging and follow-up of FDG-avid lymphomas [13].

The diagnosis of bone lymphoma is histologic. DLBCL is the most common subtype of lymphoma not only as PBL (>80%) but also as secondary invasion of the skeleton [6]. Tumor cells are large, atypical cells or a mixture of small to large cells with multilobulated nuclei and prominent nucleoli.

Immunohistochemistry analysis is positive for B cell markers CD19, CD20, and CD79a, with variable reactivity for CD10 and CD75. T cells markers are usually absent, but small CD3+ cells can be present. BCL-2 and BCL-6 immunoreactivity is variable. The Hans algorithm uses immunohistochemistry markers to further subdivide DLBCL into germinal-center B-cell (GCB) phenotype and non-GCB phenotype. This subclassification has prognostic implications [14].

Staging of PBL is mandatory as it determines prognostic and therapeutic options. The Ann Arbor modified criteria suggested by the IELSG group are usually used [3]. Stage IE implies disease in only one extranodal location (unique bone lesion). Stage IIE encompasses a single bony lesion and a regional lymphadenopathy. Stage IVE relates to multifocal disease on a single bone or lesions in multiple bones without lymph nodal or visceral disease. Stage IVE is also named “multifocal osteolymphoma” or “polyostotic lymphoma”. Finally, stage IV is used to classify disseminated lymphoma with at least one skeletal lesion. The majority of patients with bone DLBCL present with a single osseous lesion. In our patient, initial CT evaluation revealed a soft tissue mass with fracture and invasion of the ninth thoracic vertebra, suggesting a solitary bone lesion. However, imaging with MRI and PET exposed lesions also at the level of T8, T9, and T10, compatible with a polyostotic lymphoma. This highlights the importance of functional imaging when staging lymphomas.

The prognosis of patients with primary bone DLBCL is dependent on disease staging. The five-year overall survival (OS) ranges from 82% for patients with stage IE to 38% in patients with stage IV DLBCL with skeletal involvement. The traditional International Prognostic Index has predictive value in patients with disseminated DLBCL with skeletal involvement. However, it is incapable of accurate prognostic prediction in a single lesion or polyostotic PBL. The IELSG14 study found patient age, performance status, and serum LDH levels to be independently associated with OS in patients with PBL [3]. In addition, the GCB phenotype is associated with better prognosis when compared with non-GCB phenotype signatures [15]. Advanced age together with a declining performance status and non-GCB phenotype were identified as adverse prognostic markers present in our patient.

The rarity of PBL hinders the planning of randomized clinical trials. Treatment strategies are derived from retrospective studies. Approaches including chemotherapy, immunotherapy, surgery, and radiotherapy have been implemented. Surgery may be warranted to sample for histopathological diagnosis (if unsuccessful biopsy), stabilization, and internal fixation of unstable fractures, as well as decompression for spinal cord compression. In our patient, the involvement of multiple vertebrae (T8 to T10) associated with age and comorbidities justified the use of radiotherapy instead of surgery to manage spinal cord compression as described elsewhere [16].

The IELSG and the Rare Cancer Network studies suggest an anthracycline-based chemotherapy as the first-line treatment followed by field radiotherapy [3,17]. This strategy achieves an overall response rate of above 90% and a five-year OS of 84% [18]. CHOP is the preferred regimen in primary bone DLBCL with or without rituximab (R-CHOP). R-CHOP therapy does not add survival benefit, but was superior to CHOP, with three-year progression-free survival rates of 88% versus 52%, respectively [19].

The use of radiotherapy following primary chemotherapy in patients with primary bone DLBCL is controversial. Some studies show improvement, while others fail to show better outcomes when compared with chemotherapy alone [18]. These discrepancies may be due to selection biases associated with the small size of analyzed subgroups. Further investigations are necessary to validate immunochemotherapy without consolidation radiotherapy as an alternative treatment for early-stage bone DLBCL.

Patients with polyostotic lymphoma have an almost identical clinical presentation to patients with disseminated DLBCL with skeletal involvement. However, the prognosis is significantly better in patients with polyostotic DLBCL, with a five-year OS of 75% and 37% (p= 0.008), respectively [3]. The use of radiotherapy after chemotherapy in patients with polyostotic DLBCL has been associated with longer OS.

## Conclusions

This case highlighted a rare cause of low back pain due to a primary bone DLBCL. More than 90% of all vertebral tumors are metastatic disease. However, PBL should be included in the differential diagnosis of male patients above the fifth decade of life. Biopsy of the lesion is essential for accurate diagnosis. In parallel with lymphoma-related investigations, careful monitoring of possible site-associated complications, such as spinal cord compression or hypercalcemia, should be performed. Usual treatment modalities are chemotherapy with or without consolidation radiotherapy. Tailored treatment, which accounts for patient frailty, should be considered.

## References

[1] Ollila TA, Olszewski AJ (2018). Extranodal diffuse large B cell lymphoma: molecular features, prognosis, and risk of central nervous system recurrence. Curr Treat Options Oncol.

[2] Choi JH, Ro JY (2021). The 2020 WHO classification of tumors of bone: an updated review. Adv Anat Pathol.

[3] Messina C, Ferreri AJ, Govi S (2014). Clinical features, management and prognosis of multifocal primary bone lymphoma: a retrospective study of the international extranodal lymphoma study group (the IELSG 14 study). Br J Haematol.

[4] Mikhaeel NG (2012). Primary bone lymphoma. Clin Oncol (R Coll Radiol).

[5] Bindal P, Desai A, Delasos L, Mulay S, Vredenburgh J (2020). Primary bone lymphoma: a case series and review of literature. Case Rep Hematol.

[6] Messina C, Christie D, Zucca E, Gospodarowicz M, Ferreri AJ (2015). Primary and secondary bone lymphomas. Cancer Treat Rev.

[7] Beal K, Allen L, Yahalom J (2006). Primary bone lymphoma: treatment results and prognostic factors with long-term follow-up of 82 patients. Cancer.

[8] Sharma A, Ahmed R, Agrawal N (2021). Primary bone lymphoma: a 13 year retrospective institutional analysis in the chemo-immunotherapy era. Indian J Hematol Blood Transfus.

[9] Ramadan KM, Shenkier T, Sehn LH, Gascoyne RD, Connors JM (2007). A clinicopathological retrospective study of 131 patients with primary bone lymphoma: a population-based study of successively treated cohorts from the British Columbia Cancer Agency. Ann Oncol.

[10] Smith ZA, Sedrak MF, Khoo LT (2010). Primary bony non-Hodgkin lymphoma of the cervical spine: a case report. J Med Case Rep.

[11] Shen G, Su M, Liu B, Kuang A (2018). PET/CT imaging for solitary primary bone lymphoma of thoracic vertebra. Clin Nucl Med.

[12] Schaefer NG, Strobel K, Taverna C, Hany TF (2007). Bone involvement in patients with lymphoma: the role of FDG-PET/CT. Eur J Nucl Med Mol Imaging.

[13] Cheson BD, Fisher RI, Barrington SF, Cavalli F, Schwartz LH, Zucca E, Lister TA (2014). Recommendations for initial evaluation, staging, and response assessment of Hodgkin and non-Hodgkin lymphoma: the Lugano classification. J Clin Oncol.

[14] Meyer PN, Fu K, Greiner TC (2011). Immunohistochemical methods for predicting cell of origin and survival in patients with diffuse large B-cell lymphoma treated with rituximab. J Clin Oncol.

[15] Schmitz R, Wright GW, Huang DW (2018). Genetics and pathogenesis of diffuse large B-cell lymphoma. N Engl J Med.

[16] Patnaik S, Turner J, Inaparthy P, Kieffer WK (2020). Metastatic spinal cord compression. Br J Hosp Med (Lond).

[17] Cai L, Stauder MC, Zhang YJ (2012). Early-stage primary bone lymphoma: a retrospective, multicenter Rare Cancer Network (RCN) Study. Int J Radiat Oncol Biol Phys.

[18] Bruno Ventre M, Ferreri AJ, Gospodarowicz M (2014). Clinical features, management, and prognosis of an international series of 161 patients with limited-stage diffuse large B-cell lymphoma of the bone (the IELSG-14 study). Oncologist.

[19] Alencar A, Pitcher D, Byrne G, Lossos IS (2010). Primary bone lymphoma--the University of Miami experience. Leuk Lymphoma.

